# Molecular Detection of Infectious Laryngotracheitis Virus in Chickens with a Microfluidic Chip

**DOI:** 10.3390/ani11113203

**Published:** 2021-11-09

**Authors:** Mohamed El-Tholoth, Huiwen Bai, Michael G. Mauk, Eman Anis, Haim H. Bau

**Affiliations:** 1 Department of Virology, Faculty of Veterinary Medicine, Mansoura University, Mansoura 35516, Egypt; 2 Department of Mechanical Engineering and Applied Mechanics, University of Pennsylvania, Philadelphia, PA 19104, USA; hwbai@seas.upenn.edu (H.B.); mmauk@seas.upenn.edu (M.G.M.); bau@seas.upenn.edu (H.H.B.); 3 Veterinary Sciences Program, Health Sciences Division, Al Ain Men’s Campus, Higher Colleges of Technology, Al Ain 17155, United Arab Emirates; 4 New Bolton Center, Department of Pathobiology, School of Veterinary Medicine, University of Pennsylvania, Kennett Square, PA 19348, USA; eanis@vet.upenn.edu

**Keywords:** chicken, infectious laryngotracheitis virus, microfluidic device, quantitative real-time PCR, real-time LAMP

## Abstract

**Simple Summary:**

Infectious laryngotracheitis (ILT) presents a major risk to the chicken industry. Rapid, specific, simple, and point-of-need molecular detection of the virus is crucial to enable chicken farms to take timely action and contain the spread of infection. The current study describes an isothermal amplification assay for infectious laryngotracheitis virus (ILTV) infection and the implementation of this assay in a microfluidic chip suitable for molecular detection and quasi-quantification of ILTV in diagnostic veterinary laboratories with low resources and poultry farms. Our assay performance was compared and favorably agreed with quantitative PCR (qPCR). Clinical tests of our assay and chip with samples from diseased chickens demonstrated good concordance with the gold-standard benchtop qPCR assay.

**Abstract:**

Infectious laryngotracheitis (ILT) is a viral disease of chickens’ respiratory system that imposes considerable financial burdens on the chicken industry. Rapid, simple, and specific detection of this virus is crucial to enable proper control measures. Polymerase chain reaction (PCR)-based molecular tests require relatively expensive instruments and skilled personnel, confining their application to centralized laboratories. To enable chicken farms to take timely action and contain the spread of infection, we describe a rapid, simple, semi-quantitative benchtop isothermal amplification (LAMP) assay, and a field-deployable microfluidic device for the diagnosis of ILTV infection in chickens. Our assay performance was compared and favorably agreed with quantitative PCR (qPCR). The sensitivity of our real-time LAMP test is 250 genomic copies/reaction. Clinical performance of our microfluidic device using samples from diseased chickens showed 100% specificity and 100% sensitivity in comparison with benchtop LAMP assay and the gold-standard qPCR. Our method facilitates simple, specific, and rapid molecular ILTV detection in low-resource veterinary diagnostic laboratories and can be used for field molecular diagnosis of suspected ILT cases.

## 1. Introduction

Infectious laryngotracheitis virus (ILTV) causes a common respiratory disease of chickens that imposes a substantial economic load on the poultry industry. ILTV is a DNA virus that belongs to the Gallid alphaherpesvirus 1 (GaHV-1) species of the *Alphaherpesvirinae* subfamily within *Herpesviridae* family [1,2,3]. The disease causes respiratory distress and leads to significant production losses due to diminished egg production, poor feed conversion rates, high mortality rates, and increased susceptibility to other respiratory tract pathogens [2,4]. Specific and rapid diagnosis of ILT would enable implementation of timely control measures, reducing economic burdens.

Tentative diagnosis of the disease traditionally depends on clinical signs and necropsy findings. Confirmative laboratory diagnosis of ILT is performed by virus isolation, immunofluorescence techniques, neutralization assay, enzyme-linked immunosorbant assay (ELISA), as well as conventional PCR and quantitative real-time PCR (qPCR) [2,5,6,7,8,9,10,11,12,13,14]. However, the above-mentioned diagnostic methods are unsuitable for simple, specific and rapid ILTV detection outside centralized laboratories such as at rudimentary veterinary stations, and, particularly in developing countries where expensive equipment and skilled staff are available in short supply. The need to send samples to centralized laboratories and await results may delay implementation of control measures with significant adverse consequences.

Loop-mediated isothermal amplification (LAMP) of nucleic acids has been describes as a simpler alternative technology to PCR with sensitivity comparable to PCR [15,16,17,18,19]. LAMP uses a strand-displacing polymerase, obviating the need for a high-temperature ‘melting’ step and temperature cycling as used in PCR. Moreover, in contrast to PCR that uses a pair of primers, LAMP employs four primers, in addition to two loop primers, which anneal to different regions of the nucleic acid template. The additional primers may improve specificity [20].

A molecular diagnostics test (NAAT, nucleic acid amplification test) comprises two major components: (A) Enzymatic amplification and (B) Detection of the amplification product, either during amplification (real-time) or post-amplification (end-point detection). Multiple methods have been reported for amplicon detection in LAMP assays, including gel electrophoresis [21], fluorescence [21], naked eye (turbidity or color change) [21], and bioluminescence [22,23,24]. In bioluminescence and fluorescence-based detection, LAMP amplicons are monitored in real time, enabling quantification [25,26,27].

NAATs can be implemented in microfluidic formats, such as palm-sized plastic cartridges with microscale fluid circuits for sample processing and analysis [28,29]. Such microfluidic ‘chips’ afford lower cost, automated and streamlined operation, portability, sample containment, and ease of use, facilitating operation by laypeople. Multiple unit operations (e.g., lysis, nucleic extraction) can be integrated into single-use (disposable) chips for nucleic acid amplification tests [30,31].

This study describes a LAMP assay for ILTV infection and the implementation of this assay in a microfluidic chip suitable for field molecular detection and quasi-quantification of ILTV infection. Our microfluidic chip had a four reaction chambers, one of which acts as a negative control. The other three chambers can be used either to tests three different samples for the same pathogen or a single sample, split across the three chambers, for detection of co-morbidity with different pathogens.

## 2. Materials and Methods

### 2.1. Ethical Statement

Transfer of extracted nucleic acids from samples sent to the Department of Molecular Biology, School of Veterinary Medicine, University of Pennsylvania for diagnostic purpose was done according to the guidelines of the Animal Ethics Committee of School of Veterinary Medicine, University of Pennsylvania as well as the ethical guidelines of University of Pennsylvania, Philadelphia, PA, USA.

### 2.2. Chemicals and Materials

A clear resin FLGPCL04 was supplied from FormLabs^TM^ (Somerville, MA, USA). polyethylene glycol (PEG) 3350 was obtained from Sigma Aldrich, Inc. (St. Louis, MO, USA). AM1836 5× MagMax 96 Viral Extraction kit was adopted from Life Technologies™ (Ambion®, Austin, TX, USA). LAMP primers were ordered from IDT Company (Coralville, IA, USA). Loop amp DNA amplification Kit was supplied from Eiken Chemicals Co. (Tokyo, Japan). Bst 2.0 WarmStartTM DNA polymerase was purchased from New England Biolabs (Ipswich, MA, USA). EvaGreen^®^ dye was supplied from Biotium Inc. (Hayward, CA, USA). Nuclease-free water was acquired from Invitrogen (Carlsbad, CA, USA). The SsoFast EvaGreenTM Supermix for qPCR was supplied from Bio-Rad Laboratories (Bio-Rad, Hercules, CA, USA).

### 2.3. Virus and Clinical Samples

Previously isolated ILTV as well as 11 clinical samples from diseased chickens with respiratory distress were supplied by the Department of Molecular Biology, School of Veterinary Medicine, University of Pennsylvania, Philadelphia, PA, USA. The ILTV was propagated on chorioallantoic membranes (CAM) of embryonated chicken eggs (ECEs) followed by identification using histo-pathology for the intranuclear inclusions detection and the virus detected molecularly by conventional PCR [10]. The number of genomic DNA copies of ILTV was quantified as previously reported [32]. The number of genomic copies is 5 × 10^5^ copies per µL.

### 2.4. DNA Extraction

DNA/RNA extraction was carried out with AM1836 5× MagMax 96 Viral Extraction kit (Ambion^®^, Austin, TX, USA) following instructions of manufacturer.

### 2.5. LAMP Primers

**Figure 1:**
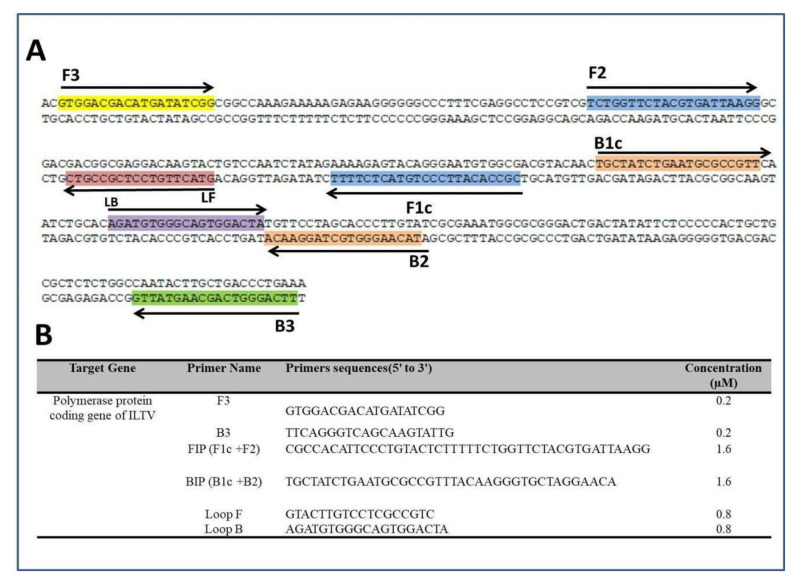
LAMP primers on the ILTV amplicon. (**A**) ILTV amplified sequence with the primers sites: forward primer (F3), backward (B3) primer, backward inner primer (BIP), forward inner primer (FIP), loop forward (LF) primer, and loop backward (LB) primer shown. Arrows and colored nucleotides show the direction of extension and the targeted sequences, respectively. (**B**) Primers sequence for ILTV LAMP assay.

Genomic sequences of various ILTV strains from the GeneBank were analyzed after alignment to identify conserved sequences. A 296-nt sequence in the polymerase gene of ILTV was used as a target due to its high similarity among the analyzed strains. The LAMP primers (Figure 1) were designed with the PrimerExplorer V5 software (Eiken Chemical Co. Ltd.). The designed primers were screened using NCBI database BLAST (http://www.ncbi.nlm.nih.gov, accessed on 2 February 2020) for cross-reaction with other chicken respiratory tract viruses, including Mareks disease virus (MDV), Newcastle disease virus and avian influenza viruses (H5N1, H9N2, and H5N8). No cross-reaction was detected. The LAMP primers were diluted to a 100 μM concentration using nuclease-free water.

### 2.6. Benchtop LAMP Amplification

The LAMP assay was performed to detect the polymerase gene of ILTV using the loop amp DNA amplification Kit. The 10 μL reaction mixture consisted of LAMP primers (Figure 1B); 5 μL of 2× Reaction Mix; 0.4 μL of Bst 2.0 WarmStart DNA polymerase; 0.5 μL 1 × EvaGreen^®^ dye (Biotium Inc., Hayward, CA, USA), and 0.6 μL of viral DNA template and nuclease-free water to 10 μL. Fluorescence signals from DNAs amplificons were observed with the 7500-Fast Real-Time PCR system (Applied Biosystems, Carlsbad, CA, USA) for 30 min at 63 °C. Template-free controls were included in each run to guarantee absence of contamination. Nucleic acid extracts of *Escherichia Coli* isolate, Newcastle disease virus (NDV) and infectious bronchitis virus (IBV) were used as negative controls.

### 2.7. Benchtop qPCR Amplification

qPCR was performed to detect the polymerase gene of ILTV using F3 and B3 primers. The 10 μL reaction mixture consisted of F3 and B3 primers (10 μM), 5 μL of SsoFast EvaGreen Supermix (Bio-Rad, Hercules, CA, USA), 0.6 μL of extracted DNA, and nuclease-free water to 10 μL. The cycling program were as follows: One cycle at 95 °C for 2 min, and then 35 cycles at 95 °C for 5 s and 60 °C for 30 s. Fluorescence emissions from amplificons was monitored with the 7500-Fast Real-Time PCR system (Applied Biosystems, Carlsbad, CA, USA). Non-template controls were included in each run. Infectious bronchitis virus (IBV), *Escherichia coli* isolate and Newcastle disease virus (NDV) nucleic acids extracts were included as negative controls.

### 2.8. Microfluidic Chip for Real-Time-LAMP

Our custom microfluidic chip (28 mm × 28 mm × 4 mm) consists of four 20 µL independent multifunctional, isothermal amplification reactors (MIAR). The microfluidic chip was designed with SolidWorks 2020 (DS SolidWorks^TM^, Waltham, MA, USA), fabricated by a Low Force Stereolithography (LFS) 3D printer Form 3 (Formlabs^TM^, Somerville, MA, USA) using a clear resin FLGPCL04 (Formlabs^TM^, Somerville, MA, USA), and coated with polyethylene glycol (PEG) 3350 aqueous solution (2%) [33].

20 μL of LAMP master mix was injected into each reactor., The reaction mix included 2 μL of the primer mix; 10 μL of 2× Reaction Mix; 0.8 μL of Bst 2.0 WarmStart DNA polymerase; 1 μL 1× EvaGreen^®^ dye, along with 1.2 μL of extracted DNA and nuclease-free water to 20 μL.

**Figure 2:**
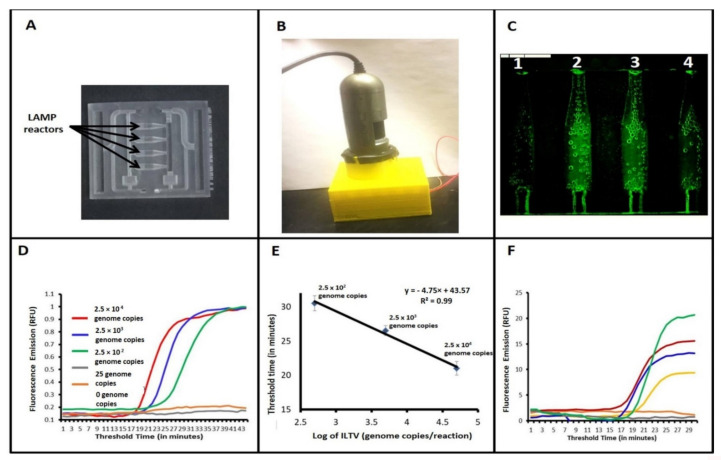
(**A**) A 3D-printed microfluidic chip with four multifunctional reactors for real-time LAMP reactions. (**B**) The portable fluorescent microscope monitors fluorescent emission from the microfluidic chip. (**C**) Image of fluorescing LAMP reactors (1: negative control; 2, 3, 4: Positive ILTV samples). (**D**) Real-time amplification curves of microfluidic chip-based ILTV LAMP assays with, 2500, 250, 25, 0 genome copies per reaction. (**E**) The microfluidic chip-based LAMP threshold time Tt (minutes) as a function of the log of ILTV concentration, (*n* = 3). (**F**) LAMP amplification curves when testing clinical samples with the microfluidic chip.

Next, we used a portable handheld fluorescence microscope (AM4113TGFBW, Dino-Lite Premier, AnMo Electronics Corp., Hsinchu, Taiwan) to monitor fluorescence signals during incubation. The microscope includes seven built-in blue light emitting diodes (LEDs) for excitation, an emission filter with a 510-nm wavelength cut-off, a CCD camera detection, and a USB connection. This microscope is appropriate for EVA Green dyes and can monitor all four reactors simultaneously without a need for scanning. The microscope is mounted on top of our custom heating system that includes a resistive heater (Figure 2B) powered by a 12V DC power adapter and regulated by a microcontroller (Raspberry Pi 4 model B, Raspberry Pi, Cambridge, UK). The chip images during incubation were taken by the fluorescence microscope once every minute and selected regions were analyzed with MATLAB (MathWorks™, R2019a, Natick, MA, USA).

The specificity of our microfluidic chip was assessed by testing various pathogens available in our laboratory: IBV (8 × 10^5^ gRNA copies per μL), NDV (10^3^ gRNA copies per μL), *E. coli* (10^10^ PFU per μL), transmissible gastroenteritis virus (TGEV, 10^3^ gRNA copies per μL), porcine epidemic diarrhea virus (PEDV, 10^3^ gRNA copies per μL), and porcine deltacoronavirus (PDCoV, 10^3^ gRNA copies per μL). Each microorganism was tested three times and presented a negative result while controls tested with appropriate primers and, otherwise, identical conditions were positive.

### 2.9. The Analytical Sensitivity

To determine the minimum copy numbers of ILTV nucleic acids that could be detected by our qPCR, LAMP, and microfluidic device, we carried out ten-fold serial dilutions of ILTV (2.5 × 10^4^ genome copies per reaction) using nuclease-free water. Each dilution was tested three times [25,26].

### 2.10. Detection of Nucleic Acids from Clinical Samples

Extraction of the nucleic acids from 11 nasal swabs collected from diseased chickens was carried out with AM1836 5× MagMax 96 Viral Extraction kit. The extracted nucleic acids were tested for ILTV by PCR as described previously [10]. The extracted nucleic acids were analyzed for ILTV with our microfluidic chip, qPCR, and real-time LAMP assays in parallel. Positive and negative controls were included in all tests.

## 3. Results

### 3.1. ILTV LAMP Performs on Par with qPCR 

**Figure 3:**
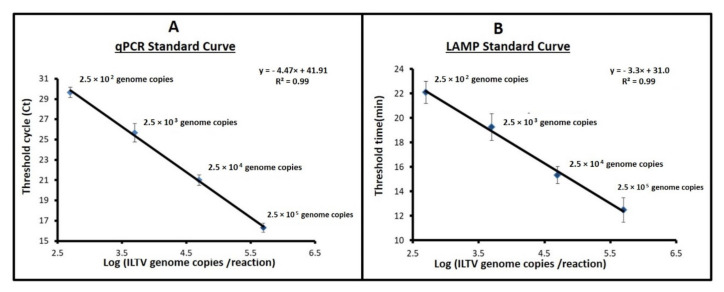
Quantitative detection of ILTV with real-time LAMP and qPCR: The PCR threshold cycle (**A**) and the LAMP threshold time (minutes) (**B**) as functions of the log of ILTV concentration (genomic DNA copies per reaction). *n* = 3.

We carried out amplification of a dilution series of ILTV with both qPCR and LAMP. As the genome concentration decreases, the threshold cycle (Ct) of qPCR increases nearly linearly as a function of the logarithm of the number of templates (Figure 3A). We define the threshold time Tt of the LAMP reaction as the time until the amplification curve reaches half its value of saturation. Like the PCR threshold cycle, the LAMP threshold time (Tt) is nearly a linear function of the log of the concentration (Figure 3B). The smallest detectable ILTV genome copies of both assays is 250 genome copies per reaction. Melting curve analysis of both the PCR and LAMP amplicons showed a single peak, confirming absence of primer-dimers and non-specific amplification. Negative controls as well as samples lacking templates did not reveal any indication of spurious amplification within 60 min of incubation.

### 3.2. Detection of ILTV with Our Microfluidic Chip and Real-Time LAMP

**Figure 4:**
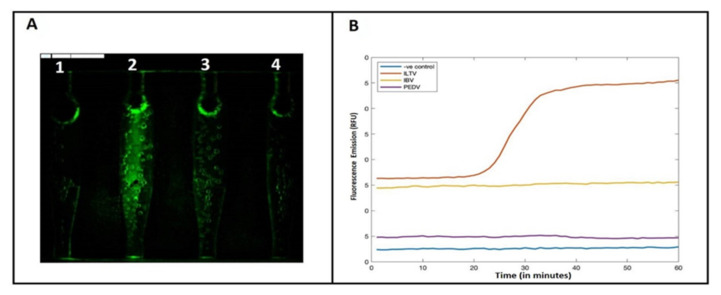
(**A**) Fluorescence emission image at the end of the LAMP amplification process in the microfluidic chip (1: negative control; 2: ILTV positive sample; 3: IBV positive sample, 4: PEDV positive sample). (**B**) Reaction chambers average fluorescence intensities as functions of time, showing positive amplification of the ILTV sample only.

The microfluidic chip used in our study has four independent MIARs (Figure 2A). Nucleic acid amplification was monitored in real time by observing fluorescence emission intensity of an intercalating dye (Figure 2B). Positive samples emitted fluorescence while the negative control did not show any fluorescence emission. When the reaction chambers achieve their incubation temperature, the fluorescence emission intensity from all reactors is low due to the absence of dsDNA. After the LAMP reaction, the first reactor on the left without any template (negative control) remains dark, consistent with the absence of amplification and dsDNA (Figure 2C). In contrast, reactors 2, 3, and 4 display fluorescence emissions, revealing successful amplification and the presence of ds DNA. The intensity of the fluorescence emission was analyzed with MATLAB software (MathWorks^TM^, R2019a) to construct amplification curves (Figure 2D). Experiments with a dilution series indicate that the threshold time correlates nearly linearly with the log of the template concentration (Figure 2E). The chip did not show any cross-reaction with IBV, NDV, *E. coli*, TGEV, PEDV and PDCoV (Figure 4). The sensitivity of LAMP in our microfluidic chip was similar to that of the benchtop LAMP (Figure 2D,E).

### 3.3. Clinical Performance of Our Assay

**Figure 5:**
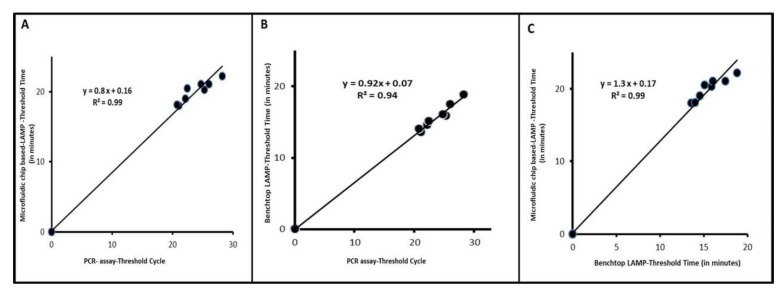
Clinical performance of our benchtop LAMP, benchtop PCR and microfluidic device when testing samples from diseased chickens (**A**) Microfluidic chip-threshold time as a function of PCR threshold cycle. (**B**) Benchtop LAMP threshold time as a function of PCR threshold cycle. (**C**) Microfluidic device threshold time as a function of benchtop LAMP threshold time.

Eleven field samples collected from diseased chicken flocks were used to screen for ILTV with our benchtop real-time LMAP and our microfluidic device. Both our benchtop and on-chip ILTV LAMP detection (Figure 2F) had 100% sensitivity and 100% selectivity in comparison with the gold-standard qPCR (Supplementary Materials, Table S1). The benchtop (Figure 5A) and microfluidic (Figure 5B) LAMP threshold times correlated nearly linearly with the qPCR threshold cycle and were shorter than the PCR processing time. The threshold times of benchtop LAMP assay and microfluidic-based RT-LAMP correlated linearly (Figure 5C). The benchtop LAMP threshold times were shorter than the microfluidic LAMP due to the higher temperature ramping rate of the former.

## 4. Discussion

ILT presents a major risk to the chicken industry [2]. Rapid and point-of-need molecular detection methods are critical to promptly instigate control measures to contain the disease. This requires samples from suspected animals to be tested in or in proximity to poultry farms. Simple molecular tests that can be carried out by minimally trained personnel and without sophisticated equipment would enable poultry farms operators to guard their farms against devastating diseases.

To address this need, we have developed a new LAMP assay for ILTV nucleic acids. LAMP assays have proven to be sensitive, specific, and robust [34,35]. LAMP assays have a few important advantages over the PCR that is used in centralized laboratories. LAMP operates at a fixed temperature (~63 ± 3 °C) and does not require temperature cycling, which diminishes equipment complexity, power consumption, and cost. Indeed, LAMP incubation can be carried out even electricity-free without any instrumentation with an exothermic reaction to provide heat and a phase change material to control incubation temperature [36]. Moreover, a previous study has demonstrated that the collective cost per LAMP reaction was roughly one third of that for PCR [37].

Furthermore, LAMP assays tolerate inhibitors effects better than PCR [38], allowing less stringent sample preparations. Finally, LAMP produces more amplicons than PCR facilitating instrument-free detection with a variety of colorimetric dyes.

Our ILTV LAMP assay can be carried out on the benchtop in a tube with standard laboratory equipment or integrated into a microfluidic device. Here, we describe a microfluidic chip with four reaction chambers (Figure 2A), one of which acts as a negative (non-template) control. The three other reaction chambers can be used to test for three different pathogens (co-morbidity) [39,40] by auto-aliquoting a single sample into multiple reaction chambers, each specialized to amplify a specific target (Supplementary Materials, Figure S1). Our microfluidic chip mates with an inexpensive, portable processor (Figure 2B) that provides both temperature control and detection. Fluorescent emission is excited and detected with a USB camera and processed with a portable device such as laptop computer. Alternatively, the chip can interface with a smartphone [41,42].

Our ILTV LAMP assay either on the benchtop or in microfluidic format performs on par with the gold-standard qPCR assay. Both our benchtop LAMP and chip assays, such as qPCR, combine amplification and detection in a closed tube/system without the need to transfer amplicons to a lateral flow strip or electrophoresis gel, avoiding exposing amplicon rich solutions to the ambient and risking possible contamination of the workplace, which may render false positives in subsequent tests. The threshold times of both our benchtop and chip-based LAMP correlate linearly with the logarithm of ILTV DNA concentration. Thus, we can use the threshold time to estimate the viral load. The somewhat longer threshold times in our microfluidic-based LAMP compared to benchtop assays result from the one-sided heating of the chip. Although in the benchtop instrument, the reaction chamber (tube) is inserted into a heated metal block, the microfluidic chip is heated only at its bottom and is exposed to the ambient at its top. The ramp up time can be, however, reduced with two-sided heating and slight increase in complexity and cost, which were deemed unnecessary.

Both our benchtop and microfluidic ILTV LAMP assays achieved sensitivity of 250 genomic copies per reaction. During early stage of infection, a chicken typically has ILTV in respiratory tissues and secretions exceeding 10^3^ genome copies/µL [12,13]. Therefore, our assays’ detection limits are more than sufficient for virus detection during the seroconversion window.

To evaluate the clinical utility of our ILTV LAMP assay and its implementation in microfluidic chip and to demonstrate that inhibitors in clinical veterinary samples do not interfere with our LAMP (both benchtop and chip), we tested 11 clinical samples from diseased chickens, demonstrating concordance with the gold-standard PCR.

Further studies for improvement of the assays for differentiating vaccine from field strains using more specific primers are needed to inform appropriate control measures. Additionally, a simple and rapid technique for pathogen nucleic acids extraction and concentration from crude samples integrated into the LAMP chip would enable higher sensitivity [43].

## 5. Conclusions

In conclusion, our newly developed real-time both benchtop and microfluidic LAMP assays enable rapid and easy detection and quasi-quantification of ILTV in diagnostic veterinary laboratories with low resources and poultry farms with sensitivity of 250 genome copies/reaction that is sufficient to detect early stages of infection. Clinical tests of our assay and chip with samples from diseased chickens demonstrated good concordance with the gold-standard benchtop qPCR assay.

## Data Availability

The data that support the findings of this study are available from the corresponding author upon reasonable request.

## Supplementary Materials

The following are available online at https://www.mdpi.com/article/10.3390/ani11113203/s1, Table S1: Results of clinical performance of our developed assays for ILTV detection, Figure S1: Two different operation modes of our generic microfluidic chip. (A) Co-detection of up to three different pathogens in one sample. (B) Testing three different samples.
