# Supplementary material for: Molecular Detection of Infectious Laryngotracheitis Virus in Chickens with a Microfluidic Chip
Source: Animals (Basel). 2021 Nov 9;11(11):3203. doi: 10.3390/ani11113203 (PMC8614514; doi:10.3390/ani11113203)
Supplement: Supplementary file 1 [file animals-11-03203-s001.zip › animals-1422389-supplementary.pdf]

animals

MDPI

Table S1. Results of clinical performance of our developed assays for ILTV detection.

Samples No.	ILTV Detection		
	qPCR (Ct)	LAMP (Min)	Microfluidic Chip (Min)
1	21.06	13.56	18.01
2	0.00	0.00	0.00
3	26.00	17.46	21.40
4	22.13	14.55	19.01
5	0.00	0.00	0.00
6	25.23	15.85	20.30
7	22.43	15.1	20.51
8	20.75	14.01	18.01
9	28.21	18.8	22.18
10	0.00	0.00	0.00
11	24.72	16.02	21.04
-ve control	0.00	0.00	0.00

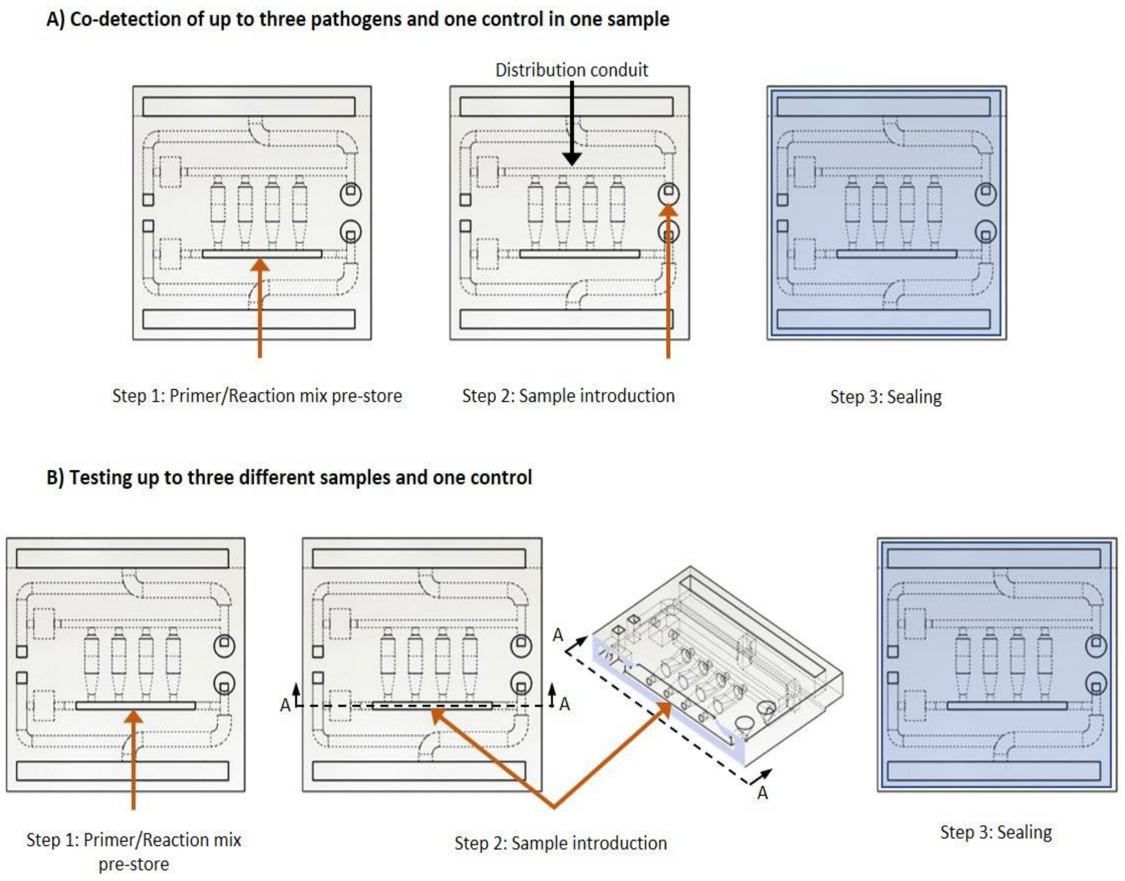

Figure S1. Two different operation modes of our microfluidic chip. **(A)** Co-detection of three different pathogens in a single sample and a control. Step 1 – Primer/reaction mix pre-store: Dried target-specific primer sets are pre-stored in the individual reaction chambers during chip fabrication. Although not attempted here, dried LAMP reaction mix can also be pre-stored in either the distribution conduit or in the individual reaction chambers. Step 2 – Sample introduction: Templates suspended in water (when the LAMP reaction mix is pre-stored) or in LAMP reaction mix (absenting primers, when the LAMP mix is not pre-stored) are introduced into the distribution conduit, flow into the various reaction chambers by capillary action, and are halted by passive capillary valves located at the distal ends of the reaction chambers. Step 3 – Sealing: – The chip is sealed with adhesive tape and is ready for incubation. **(B)** Testing three different samples and a control. Step -1 primers and reaction mixes are dried in the various reaction chambers. Step 2 – Different samples are pipetted into individual reaction chambers, hydrating the dried reagents. The samples are retained in the reaction chambers with passive (capillary) valves. Step 3 – Sealing: – The chip is sealed with adhesive tape and is ready for incubation.
